# New bone ingrowth into β-TCP/HA graft activated with argon plasma: a histomorphometric study on sinus lifting in rabbits

**DOI:** 10.1186/s40729-020-00236-4

**Published:** 2020-08-13

**Authors:** Kazushige Tanaka, Daniele Botticelli, Luigi Canullo, Shunsuke Baba, Samuel P. Xavier

**Affiliations:** 1 Department of Oral Implantology, Osaka Dental University, Osaka, Japan; 2 ARDEC Academy, Rimini, Italy; 3 Department of Stomatology, Faculty of Medicine and Dentistry, University of Valencia, Valencia, Spain; 4 Department of Oral Implantology, Osaka Dental University, Osaka, Japan; 5 Depto CTBMF e Periodontia FORP-USP-Faculty of Ribeirão Preto (SP), São Paulo, Brazil

**Keywords:** Animal study, Sinus floor elevation, Bone healing, Xenograft, Argon plasma

## Abstract

**Background:**

In a previous experimental study, new bone was found growing within granules of HA/β-TCP. In vitro and experimental studies have shown increased protein adsorption and cell adhesion graft material bioactivated with Argon plasma. The aims of the present experiment were to study new bone ingrowth into β-TCP/HA granules used as filler material for sinus lifting and the influence on the healing of the bioactivation of the graft with argon plasma.

**Methods:**

Sinus lifting was carried out in 20 rabbits using 60% HA and 40% β-TCP as filler material either bio-activated with argon plasma (plasma group) or left untreated (control group). The antrostomies were closed with collagen membranes. Biopsies representing the healing after 2 and 10 weeks were collected, and ground sections were prepared for histomorphometric analyses. Various regions of the elevated space were analyzed both around (outer bone; OB) and inside (interpenetrating bone network, IBN) the graft particles.

**Results:**

After 2 weeks of healing, 8.2% and 9.3% (*n* = 10; *p* = 0.635) of total new bone (OB + IBN) was found in the plasma and control groups, respectively. Small fractions of IBN were found, spreading from the periphery inward of the graft particles. After 10 weeks of healing, the total new bone was 34.0% in the plasma and 31.3% in Control groups (*n* = 9; *p* = 0.594). The respective fractions of IBN were 18.0% and 16.0%. New bone was penetrating from the peripheral regions inside the remnants of graft particles, where it was forming a network of bridges in continuity to the remnants of biomaterial through its porosities. The biomaterial decreased in proportion between 2 and 10 weeks from 52.1 to 28.3% in the plasma group, and from 52.5% to 31.9% in the control group.

**Conclusion:**

The bio-activation with argon plasma on a synthetic graft composed of 60% HA and 40% β-TCP used as filler material for sinus lifting showed a tendency to improve bone formation; however, the difference with the control group was neither statistically significant nor clinically relevant.

## Introduction

The use of biomaterials to maintain the volume obtained after sinus floor elevation is a procedure well documented in scientific literature [1–3]. Depending on the degrees of resorption of the graft used, different proportions of bone might be found inside the elevated space. In a systematic review with meta-analysis [4], it was shown that the autologous bone alone produced the highest amount of new bone when compared to xenografts or synthetical biomaterials.

A literature review [5] showed the intrinsic healing pattern of this anatomical structure, underlining the importance of the bone walls of the sinus cavity, which were recognized to be the most important source of blood supply and regenerative potential.

The histologically pattern of this clinical phenomenon was clarified in several animal studies [6–14] These studies in fact confirmed that the regeneration process starts from the bony walls and increases if the lateral bony wall is maintained attached to the mucosa [15, 16]..

Although the importance of the graft material to fill the sinus cavity was questioned by a recent systematic review [17] highlighting the possibility to longitudinally maintain implants in a sinus lifted without graft, however a significant difference in terms of survival rate was found when compared to grafted sinus.

Although the best material was documented to be the autologous bone, due to its osteo-inductive properties, it was documented that the use of a graft material, mostly in case of critical anatomical conditions, could speed and enhance the quality of hard tissue regeneration under the sinus mucosa [18].

All the graft materials, however, share the same biologic path to be osseointegrated: the key factor is represented by their wettability once exposed to the blood proteins. In fact, linking proteins (extracellular matrix molecules) are essential for the initiation and modulation of cell adhesion with regenerative potential [19]. Then, the material properties, moreover the wettability of the graft granules, may represent a key factor in bone regeneration.

One technique recently appeared on the literature to increase the graft surface hydrophilicity, is plasma of Argon which through the alteration of the electronic mantel of the surface, positively alter the surface charge of the material [20]. In fact, the treatment with argon plasma has been tested in an in vitro study in which four types of discs made of synthetic pure hydroxyapatite, biphasic calcium phosphate (60% HA, 40% β-TCP), cancellous and cortical xenogeneic bone matrix were used [21]. It was shown that the bioactivation increased significantly protein adsorption and cell adhesion. Plasma treatment has been shown to increase also the osteoconductivity on biomaterials [22] and osseointegration on implants [23].

Moreover, a recent publication showed that bovine bone matrix in the rabbit sinus lift clearly identified a significantly better regeneration pattern in the central area of the sinus in case of bioactivated graft, the most distant area from osteogenesis sources [24].

It was furthermore described in an experimental study in sheep the pattern of healing of 40% β-TCP/60% HA granules used for sinus lifting. Large amounts of new bone were found growing inward the synthetic biomaterial, interpenetrating the resorbing graft granules [25].

Hence, the aims of the present experiment were to study new bone ingrowth into β-TCP/HA granules used as filler material for sinus lifting and the influence on the healing of the bioactivation of the graft with argon plasma. The hypothesis was that the treatment with argon plasma might enhance bone formation both around and within of the HA and β-TCP granules.

## Materials and methods

### Ethical statement

The ethical approval of the protocol for the present study was given by the Ethical Committee at the School of Dentistry, of Ribeirão Preto, University of Sao Paulo (USP), with the code 2018.1.454.58.2 signed on 19 September 2018. The ARRIVE checklist for animal studies was followed. The international and local guidelines for animal experiments were respected.

### Study design

Sinus floor augmentation was performed bilaterally in 20 rabbits. The elevated spaces were grafted with a synthetic biomaterial either activated (plasma group) or not activated (control group) with argon plasma. Ten rabbits were euthanized after 2 weeks and 10 rabbits after 10 weeks.

### Bioactivation with argon plasma

The alloplastic granules were moved from the vials to a sterile cup using a small spoon and then placed in an argon plasma reactor (10 W, 1 bar for 12 min, plasma R, Diener, Germany) for the activation procedure.

### Experimental procedures

The sedation was performed using acepromazine 1.0 mg/kg (Acepran®, Vetnil, Louveira, São Paulo, Brazil) injected subcutaneously, followed by the anesthesia that was carried out with xylazine 3.0 mg/kg (Dopaser®, Hertape Calier, Juatuba, Minas Gerais, Brazil) and ketamine hydrochloride 50 mg/kg (Ketamin Agener, União Química Farmacêutica Nacional S/A, Embu-Guaçú, São Paulo, Brazil) IM. Local anesthesia was also carried out with mepivacaine 2% and epinephrine 1:100.000 (Mepiadre, Nova DFL, Rio de Janeiro, Brazil).

**Fig. 1:**
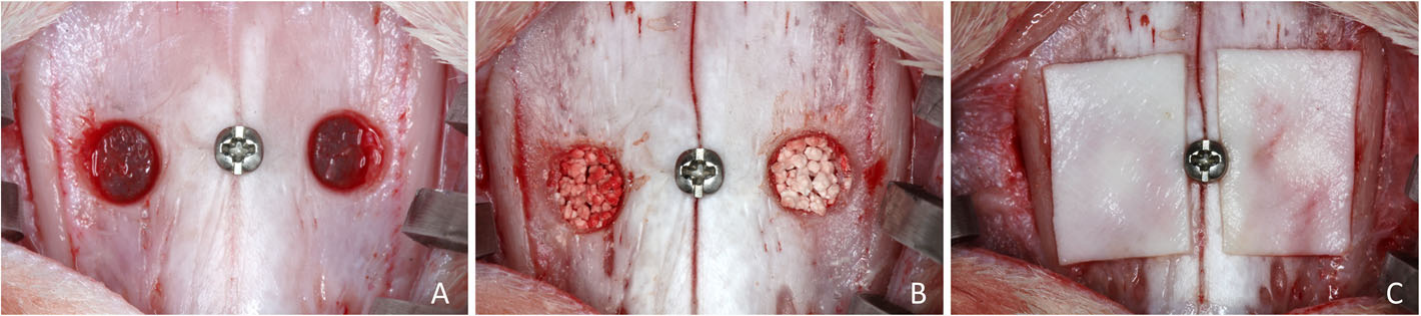
Clinical view of the surgical procedures. **a** Rounded antrostomies were prepared with a trephine 3.5 mm in diameter, both sides of the nasal-incisal suture. **b** After the elevation of the sinus mucosa, dried 60% hydroxyapatite and 40% beta-tricalcium phosphate granules were grafted into the elevated space (plasma site on the left)

In all rabbits, a masked maxillofacial surgeon (ERS; see the “Acknowledgements” section) performed a dermo-periosteal incision, exposed the nasal bone, and prepared rounded antrostomies with a trephine 3.5 mm in diameter, both sides of the nasal-incisal suture (Fig. 1a). The bone window was removed and the sinus mucosa was elevated and then grafted with similar amounts of biomaterial (~ 130 ml), composed of 60% hydroxyapatite and 40% beta-tricalcium phosphate irregular-shaped granules (GUIDOR calc-i-oss CRYSTAL^+^; Sunstar, Etoy, Switzerland), with dimension of 450–1000 micrometers (Fig. 1b). The granules contained macropores and only those used for the test sites (plasma group) were bioactivated in the argon plasma reactor. Both for the plasma and control groups, the granules were maintained dried until the placement in the elevated space. Collagen membranes (Bio-Gide; Geistlich Biomaterials, Wolhusen, LU, Switzerland) were used to cover both antrostomies (Fig. 1c).

### Euthanasia

After sedation, the animals were euthanized with sodium thiopental (1.0 g, 2 mL, Thiopentax®, Cristália Produtos Químicos Farmacêuticos, Itapira, São Paulo, Brazil).

### Experimental animals

Twenty New Zealand male rabbits of about 5–6 months of age and 3–3.5 kg of weight used for the experiment.

### Housing and husbandry

The animals were maintained individually in cages located in rooms under a controlled environment at the facilities of the School of Dentistry, USP, Ribeirão Preto (Brazil). Pain, biological functions and wounds were daily monitored. No restrictions were applied for food and water.

The animals received a prophylactic dose of oxytetracycline dehydrate (40 mg/kg, IM, Terramicina LA, Zoetis Indústria e Produtos Veterinários, Campinas, Sao Paulo, Brazil). Postoperatively, the animals received ketoprofen (3.0 mg/kg, IM., Ketofen 1%, Merial, Monte-Mor, Sao Paulo, Brazil) and tramadol hydrochloride (Tramadol 2%, 1.0 mg/kg, SC., Cronidor, Agener União Saúde Animal, Apucarana, Parana, Brazil) for 2 days.

### Sample size

No data were available on similar treatment in vivo on alloplastic graft with argon plasma so that a difference of 10% of new bone within the elevated space was judged as clinically relevant. Applying a standard deviation of 10%, a power of 0.8 and an α = 0.05, a sample of 10 animals each group was calculated to disclose differences clinically relevant. This allowed maintaining the number of animals as low as possible, as required by the 3R rules [26].

### Randomization and allocation concealment

The randomization was made by a researcher that did not participate to the surgery (DB) at the website www.randomization.com. The allocation treatments were secured in sealed opaque envelopes that were opened after the elevation of both sinuses by an author (SPX) not involved in the surgical procedures.

### Histological preparation and analyses

The histological procedures were already described in a previous article [10]. Briefly, the sections, each containing both sinuses, were prepared in blocks, fixed in 10% buffered formalin, dehydrated in ascending concentrations of alcohol, and then embedded in resin. Two ground sections were prepared for each biopsy using the Exakt equipment (Exakt®, Apparatebau, Norderstedt, Germany). The histological slides were stained with either Stevenel’s blue and alizarin red or toluidine blue. Digital photomicrographs of all ground sections were taken at a magnification × 100 using an EK14 motorized stage (Nikon Corporation, Tokyo, Japan) connected to an Eclipse Ci microscope (Nikon Corporation, Tokyo, Japan).

Morphometric evaluations were performed in the histological slides stained with Stevenel’s blue and alizarin red. The software NIS-Elements D 5.11 (Laboratory Imaging, Nikon Corporation, Tokyo, Japan) was used for measurements. For this purpose, a lattice with squares of 75 μm in dimensions was superposed to the images. Various regions of the sinus were evaluated: (i) close to the bone walls (Bone walls region), (ii) the most central area of the elevated space (Central region), (iii) the region subjacent the sinus mucosa (Sub-mucosa region), (iv) and a region close to the antrostomy (close-to-window region). The following tissues were assessed: outer bone (OB; new bone outside the biomaterial residues), interpenetrating bone network (IBN; new bone consolidated within the biomaterial residues), residual alloplastic graft, soft tissues, vessels, osteoclastic zones, and inflammatory infiltrate.

### Calibration for histometric evaluations

An expert examiner, not included in the list of authors, did all histological assessments (KAAA see the “Acknowledgements” section), after having performed a calibration with another expert (DB) that resulted in a *K* > 0.90 inter-rater agreement.

### Experimental outcomes

The primary variables were the percentage of the interpenetrating bone network (IBN) and the percentage of total bone within the elevated space that was calculated as the sum of the outer bone (OB) and the interpenetrating bone network (IBN). The other variables, namely, outer bone, residual alloplastic graft, soft tissues, vessels, osteoclastic zones, and inflammatory infiltrate were considered as secondary variable.

### Statistical methods

Differences between plasma (test) and the control groups were analyzed with a Wilcoxon test using the software IBM SPSS Statistics (IBM Inc., Chicago, IL, USA). An α = 5% was applied.

## Results

None of the animals presented complications at the surgery and during the maintenance period. However, one histological specimen belonging to the 10 weeks group was lost for technical problems so that *n* = 10 and an *n* = 9 were achieved for the 2-week and 10-week groups, respectively.

**Fig. 2:**
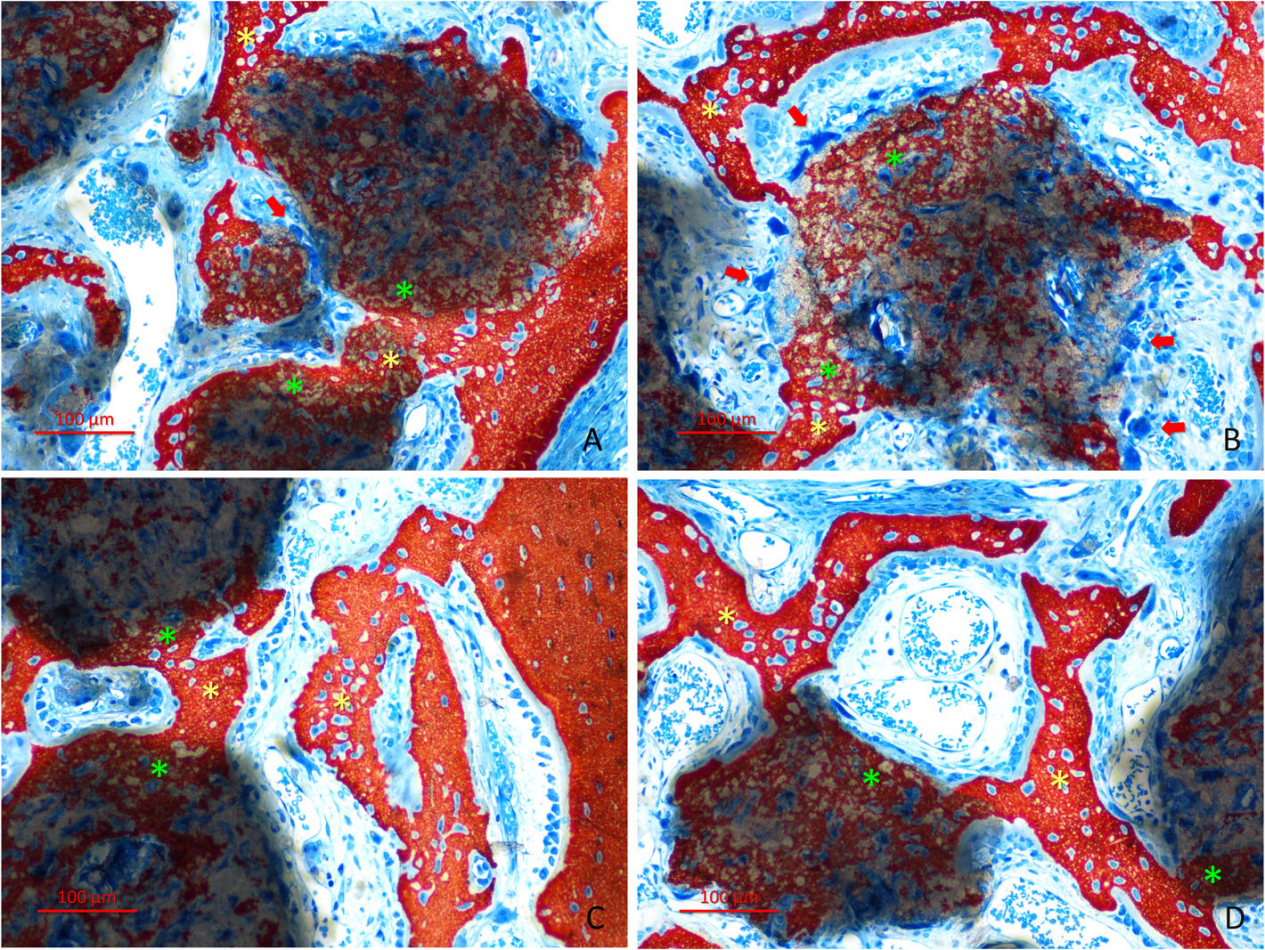
Graft particles showing various grades of resorption after 2 weeks of healing. New bone was found surrounding the graft particles in the periphery (outer bone; OB) and spreading inward the graft particles in small quantities (interpenetrating bone network; IBN). Soft tissues, cells and vessels are visible within the graft. Osteoclastic zones were found in the periphery of the graft (red arrows in **a** and **b** indicate some examples). **a**, **b** Plasma group. **c**, **d** Control group. **a**–**c** Bone wall regions. **b**–**d** Sub-mucosa regions. The asterisks indicate examples of OB (yellow) and IBN (green). Stevenel’s blue and alizarin red stain

After 2 weeks of healing (Fig. 2a–d), in both groups, new bone was found surrounding the graft particles in the periphery (outer bone, OB) and spreading inward the graft particles in small quantities (interpenetrating bone network, IBN). Soft tissues, cells and vessels were visible both around and within the graft. Osteoclasts were found in the periphery of the biomaterial. The total new bone was 8.2 ± 7.0% and 9.3 ± 8.5% at the plasma and control group, respectively (*p* = 0.635; Table 1). Most of the new bone was located outside (OB) the residues of the granules of biomaterial. However, few fractions of 2.2 ± 2.4% and 3.3±4.3% (p=0.093) of new bone was found inside the residues of the granules (IBN). Biomaterial devoid of new bone was found in proportion of 52.1 ± 12.4% in the plasma group, and 52.5 ± 8.1% in the control group (*p* = 0.575).

**Table 1:** Tissues components in percentages (%) in the elevated area in the plasma and control sites after 2 and 10 weeks of healing. Mean values (in bold) ±standard deviation and 95% confidence interval (upper; lower)

	OB	IBN	Total new bone	β-TCP HA	Soft tissues	Vessels	Osteoclastic zones	Inflammatory infiltrate
2 weeks plasma	**6.1** ± 4.9 (3.0; 9.1)	**2.2** ± 2.4 (0.7; 3.6)	**8.2** ± 7.0 (3.9; 12.6)	**52.1** ± 12.4 (44.4; 59.7)	**37.4** ± 8.4 (32.2; 42.5)	**2.2** ± 1.3 (1.4; 3.0)	**0.1** ± 0.2 (0.0; 0.1)	**0.1** ± 0.4 (0.0; 0.4)
2 weeks control	**6.0** ± 5.0 (2.9; 9.1)	**3.3** ± 4.3 (0.6; 6.0)	**9.3** ± 8.5 (4.0 16.4)	**52.5** ± 8.1 (47.5; 57.5)	**35.6** ± 5.9 (31.9; 39.3)	**2.6** ± 1.1 (1.9; 3.3)	**0.0** ± 0.1 (0.0; 0.1)	**0.0** ± 0.0 (0.0; 0.0)
*p*	0.594	0.093	0.635	0.575	0.507	0.069	0.180	0.317
10 weeks plasma	**16.0** ± 9.2 (9.9; 22.0)	**18.0** ± 9.0 (12.2; 23.9)	**34.0** ± 17.3 (22.7; 45.4)	**28.3** ± 12.6 (20.1; 36.5)	**35.7** ± 8.0 (30.5; 41.0)	**1.9** ± 0.7 (1.4; 2.3)	**0.1** ± 0.1 (0.0; 0.1)	**0.1** ± 0.2 (− 0.1; 0.2)
10 weeks control	**15.3** ± 5.1 (12.0; 18.7)	**16.0** ± 8.3 (10.6; 21.5)	**31.3** ± 11.9 (23.5; 39.1)	**31.9** ± 11.8 (24.2; 39.6)	**34.6** ± 5.4 (31.3; 38.1)	**1.9** ± 1.0 (1.2; 2.5)	**0.2** ± 0.3 (0.0; 0.3)	**0.1** ± 0.2 (0.0; 0.2)
*p*	0.767	0.407	0.594	0.314	0.678	0.726	0.416	0.317

*OB* outer bone, *INB* interpenetrating bone network. Total new bone = OB + INB. *β-TCP HA* residual alloplastic graft.

None of the difference was statistically significant

Within the various regions examined, the highest amount of new bone was found in the Bone walls region while the lowest amount was in the central region (Table 2). Most commonly, higher amounts of OB were found compared to IBN. The highest fractions of IBN (~ 5%) were found in the Bone walls regions of both groups and in the Sub-mucosa region of the control group. No differences were found between groups for any of the variables evaluated.

**Table 2:** Hard tissue components in percentages (%) in the various regions of the elevated area in the plasma and control sites after 2 weeks of healing. Mean values (in bold) ±standard deviation and 95% confidence interval (upper; lower)

	OB	IBN	Total new bone	β-TCP HA
Bone walls plasma	**12.1** ± 6.4 (8.1; 16.1)	**5.2** ± 5.5 (1.8; 8.6)	**17.3** ± 10.7 (10.6; 23.9)	**42.7** ± 9.7 (36.7; 48.8)
Bone wall control	**11.5** ± 7.8 (6.7; 16.4)	**5.1** ± 5.7 (1.6; 8.7)	**16.6** ± 11.7 (9.4; 23.9)	**46.5** ± 11.8 (39.2; 53.8)
*p*	0.646	0.635	0.575	0.169
Central plasma	**2.1** ± 3.1 (0.2; 4.1)	**1.0** ± 1.8 (− 0.1; 2.1)	**3.1** ± 4.1 (0.6; 5.7)	**58.0** ± 15.1 (48.6; 67.3)
Central control	**2.1** ± 2.7 (0.5; 3.8)	**1.9** ± 3.4 (− 0.2; 4.0)	**4.0** ± 5.7 (0.5; 7.6)	**59.9** ± 9.4 (54.1; 65.7)
*p*	1.000	0.465	0.345	0.721
Sub-mucosa plasma	**6.9** ± 9.3 (1.2; 12.6)	**2.4** ± 2.9 (0.6; 4.1)	**9.3** ± 11.9 (1.9; 16.7)	**55.1** ± 13.7 (46.6; 63.6)
Sub-mucosa control	**4.0** ± 4.5 (1.2; 6.8)	**5.3** ± 6.9 (1.0; 9.6)	**9.4** ± 11.4 (2.3; 16.4)	**51.7** ± 13.3 (43.4; 59.9)
*p*	0.310	0.075	0.866	0.284
Close-to-window plasma	**3.3** ± 4.5 (0.5; 6.1)	**0.6** ± 2.0 (− 0.6; 1.9)	**4.0** ± 6.3 (0.1; 7.9)	**56.3** ± 15.3 (46.8; 65.8)
Close-to-window control	**5.2** ± 5.4 (1.9; 8.6)	**2.5** ± 3.6 (0.3; 4.7)	**7.7** ± 8.0 (2.8; 12.7)	**52.6** ± 14.9 (43.4; 61.9)
**p**	0.271	0.068	0.128	0.767

*OB* outer bone, *INB* interpenetrating bone network. Total new bone = OB + INB. *β-TCP HA* residual alloplastic graft

None of the difference was statistically significant.

**Fig. 3:**
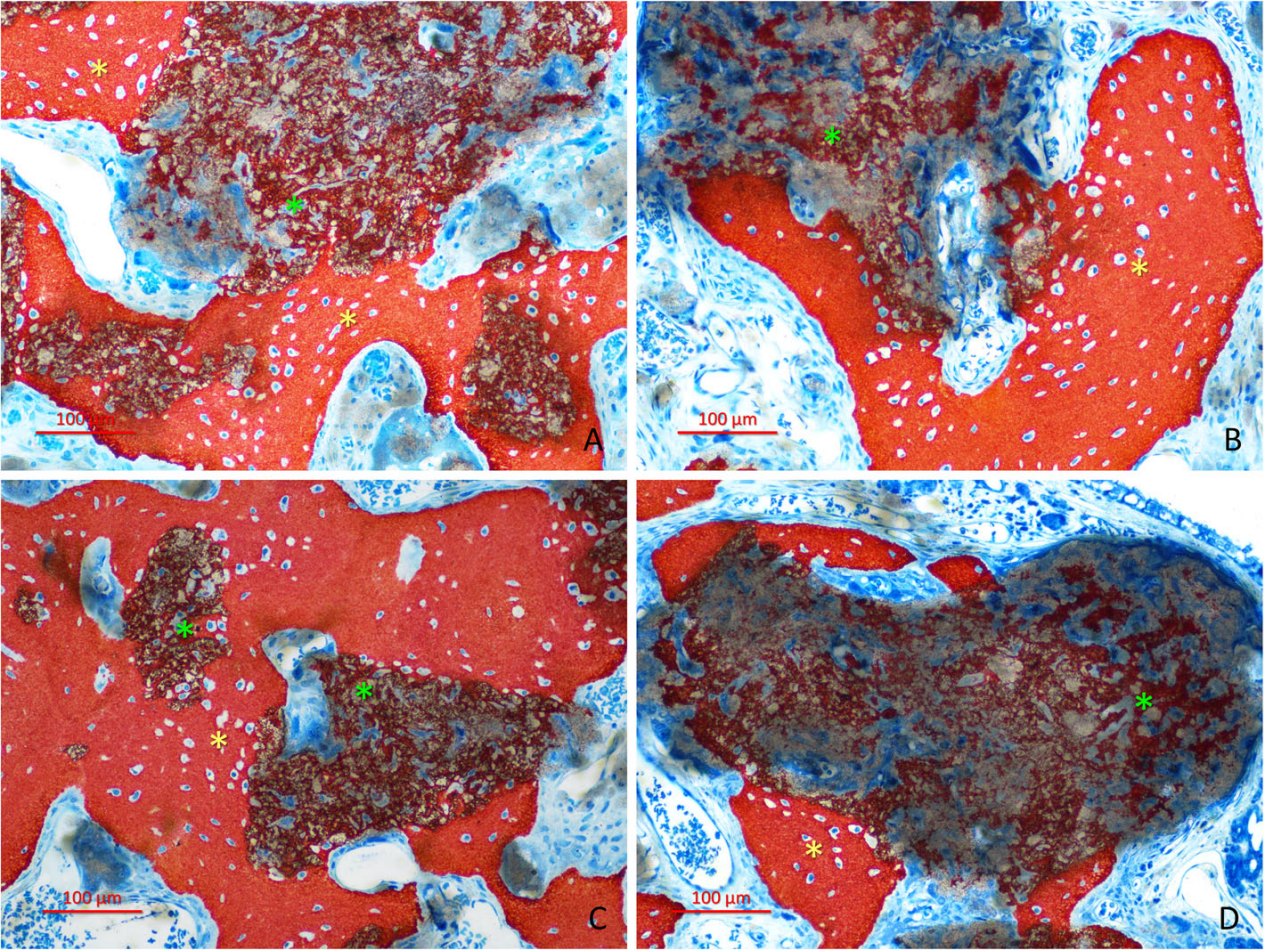
Healing after 10 weeks. Higher amounts of newly formed bone were observed in this period of healing compared the previous period analyzed, in both the plasma group (**a**, **b**) and the control group (**c**, **d**). New bone was penetrating from the peripheral regions (outer bone; OB) inside the remnants of graft particles forming a network of bridges interconnecting the peripheral bone with the biomaterial through its porosities (Interpenetrating bone network; IBN). **a** Central region. **b**–**d** Sub-mucosa regions. **c** Next to the bone wall region. The asterisks indicate examples of OB (yellow) and IBN (green). Stevenel’s blue and alizarin red

After 10 weeks of healing, in both groups, higher amounts of newly formed bone were observed compared to the previous period analyzed. Biomaterial was still present, but in lower percentages compared the 2-week period. New bone was penetrating from the peripheral regions inside the remnants of graft particles forming a network of bridges interconnecting the outer bone with the biomaterial through its porosities (Fig. 3 a-d). Within the elevated space, new bone increased to 34.0 ± 17.3% in the plasma group and to 31.3 ± 11.9% in the control group (*p* = 0.594; Table 1). In this stage on healing, slightly higher amounts of IBN within the granule residues were found compared to the OB formed outside of these residues. No statistically significant differences were found between groups for both the OB (*p* = 0.767) and IBN (*p* = 0.407).

Within the various regions examined, the highest amount of new bone was found again in the bone walls region, reaching proportions of 47.1 ± 12.4% and 41.9 ± 12.5% in the plasma and control group, respectively (*p* = 0.214; Table 3). The new bone increased in all regions examined and higher amounts of IBN were found compared to OB in all regions with the exclusion of the Bone walls region.

**Table 3:** Hard tissue components in percentages (%) in the various regions of the elevated area in the plasma and control sites after 10 weeks of healing. Mean values (in bold) ±standard deviation and 95% confidence interval (upper; lower)

	OB	IBN	Total new bone	β-TCP HA
Bone walls plasma	**25.1** ± 7.5 (20.1; 30.0)	**22.0** ± 6.8 (17.5; 26.5)	**47.1** ± 12.4 (39.0; 55.1)	**20.1** ± 10.1 (13.5; 26.8)
Bone walls control	**24.4** ± 8.5 (18.9; 29.9)	**17.5** ± 8.7 (11.8; 23.2)	**41.9** ± 12.5 (33.7; 50.1)	**25.0** ± 11.9 (17.2; 32.8)
*p*	0.859	0.051	0.214	0.051
**Central plasma**	**11.8** ± 11.6 (4.2; 19.4)	**21.4** ± 11.2 (14.1; 28.8)	**33.2** ± 19.6 (20.4; 46.0)	**29.4** ± 18.6 (17.2; 41.6)
**Central control**	**5.9** ± 6.5 (1.6; 10.1)	**17.9** ± 13.3 (9.2; 26.6)	**23.8** ± 19.1 (11.3; 36.2)	**38.9** ± 15.0 (29.1; 48.7)
*p*	0.161	0.362	0.208	0.214
**Sub-mucosa plasma**	**12.2** ± 11.7 (4.5; 19.8)	**12.4** ± 11.8 (4.7; 20.1)	**24.6** ± 20.5 (11.2; 38.0)	**38.1** ± 20.0 (25.0; 51.2)
Sub-mucosa control	**8.5** ± 11.9 (0.7; 16.3)	**12.8** ± 11.7 (5.1; 20.4)	**21.2** ± 20.8 (7.7; 34.8)	**40.9** ± 21.4 (26.9; 54.9)
*p*	0.515	0.678	0.594	0.678
Close-to-window plasma	**12.9** ± 11.9 (5.1; 20.7)	**15.9** ± 8.5 (10.4; 21.5)	**28.8** ± 17.6 (17.3; 40.3)	**29.4** ± 12.2 (21.4; 37.4)
Close-to-window control	**13.4** ± 8.4 (7.9; 18.9)	**14.5** ± 9.7 (8.1; 20.8)	**27.9** ± 15.9 (17.5; 38.3)	**29.8** ± 16.4 (19.1; 40.5)
*p*	0.767	0.594	0.813	0.906

*OB* outer bone, *INB* interpenetrating bone network. Total new bone = OB + INB. *β-TCP HA* residual alloplastic graft

None of the difference was statistically significant

## Discussion

The aims of the present experiment were to study new bone ingrowth into β-TCP/HA granules used as filler material for sinus lifting and the influence on the healing of the bioactivation of the graft with argon plasma.

Small fractions of interpenetrating bone network (IBN) were already present after 2 weeks of healing. After 10 weeks, IBN reached similar percentages of the outer bone (OB).

After 2 weeks of healing, similar amounts of new bone were found in the plasma (8.2%) and in the control sites (9.3%). After 10 weeks of healing, bone increased considerably in both groups, with a tendency of higher bone formation in the plasma (34.0 ± 17.3%) compared to the control group (31.3 ± 11.9%). However, no statistically significant differences were found both after two (*p* = 0.635) and 10 (*p* = 0.594) weeks of healing.

The results from the present study are in agreement with those from another similar study in which DBBM granules were used [24]. In that study, similarly to the present study, the granules of the biomaterial planned to be grafted into the sinus of the test sites were activated with argon plasma. After 2 weeks of healing, similar fractions of woven bone were detected in both test (5.2%) and control (5.0%) sites. In the present study, after 2 weeks of healing, slightly higher values of new bone were found compared to that of the study mentioned above. These higher values might be related to that fact that bone was also formed inside the remnants of biomaterial (interpenetrating bone network) so that the total amount of new bone was expressed as the sum of the IBN and the outer bone (OB) formed outside the remaining graft.

In the study mentioned above [24], only the bone outside the granules of DBBM was assessed. The bone that possibly grew within the DBBM was not visible at the histological examination maybe because of its reduced quantity due to the high density, the low rate of resorption, and the low size of the porosities of that biomaterial [9, 27, 28]. In the present study, slightly over-lighting the histological slides at the light microscope, the new bone turned out to be visible through the graft under resorption. This allowed an evaluation also of this bone (IBN) that was penetrating inside the graft, forming a network of bridges interconnecting the outer bone with the remnants of the porous biomaterial. This aspect had been already described in a previous experimental study on sinus lifting in sheep [25]. After sinus mucosa elevation, the elevated space was filled with a similar graft used in the present study, also composed of 60% HA and 40% β-TCP. At the test sites, the bone window was repositioned on the antrostomy while, at the control site, the antrostomy was protected with a citric acid ester membrane. The graft was found interpenetrated by new bone for 37.1% and 33.1% at the test and control sties, respectively. The outer bone outside the graft was 16.4% and 15.0%, respectively.

After 10 weeks of healing, in a study mentioned above [24], 23.5% and 21.3% of new bone was found at the plasma and control groups, respectively. These proportions were higher compared to those of the OB assessed outside the graft granules in the present study that were 16.0% and 15.3% in the plasma and control group, respectively. However, in the present study, the IBN contributed to increase the total amount of new bone to 34.0% in the plasma group and 31.3% in the control group.

It should be considered that the biomaterial used in the present experiment exhibited macroporosity and a higher degradation compared to the rate of resorption reported for the DBBM. The intrinsic characteristics of the synthetic biomaterial used in the present study allowed the new bone to invade part of the graft while it was resorbing. In the DBBM study, instead, the new bone was laying on the surface of the graft. A larger portion of the elevated space was still occupied by this biomaterial after 10 weeks of healing compared to that found in the present study. In fact, even though, after 2 weeks of healing, the proportion of biomaterial was higher in the present experiment by 3–4% compared to the DBBM study [24]; after 10 weeks, the percentages of HA/β-CTP turned out to be lower compared to the DBBM percentage. This, in turn, means that the DBBM granules were still occupying larger part of the elevated space of the sinus, allowing the bone to grow on their surface and within the zones interposed among granules. Instead, the higher rate of graft degradation of the HA/β-TCP in conjunction with the growth of bone inside the macroporosities of the graft provided a further environment into which the bone could grow. This might explain the higher amount of new bone found in the present experiment compared to that reported in the DBBM study.

Bone formation in sinuses lifted using DBBM has been described in several studies [9, 14, 29, 30]. It was reported that new bone is mainly formed from the sinus bone walls and then propagates within the elevated space, towards the other regions. The DBBM granules are initially surrounded by a dense tissue rich in mesenchymal cells that, over time, is substituted by new bone contributing to the consolidation of the graft into the newly formed bone [9].

Similar experiments in rabbits used a collagenated cortico-cancellous porcine bone that presented a different pattern of healing compared to the present study [10–13, 31]. In one of these experiments [12], the bony window was repositioned on the antrostomy at the test site while, at control site, a collagen membrane was applied. After 2 weeks of healing, ~ 2% of new bone and 40% of xenograft were found in both sites. About 3% of resorption zones containing multinucleated cells were identified while, in the present study, only 0.1–0.2% of these cells was found. After 8 weeks of healing, new bone reached proportions of ~ 23–24% and the xenograft was reduced to ~ 11%. About 2% of resorptive zones with multicellular units were still observed. The collagenated cortico-cancellous porcine bone was either resorbed before allowing bone formation or, similarly to the DBBM, it was enclosed into new bone formed on its surface. No bone within the graft remnants was identified.

In the present study, after 10 weeks of healing, only 2.7% higher amount of total new bone was found at the plasma compared to the control group. The highest difference (9.4%; *p* = 0.208) in favor of the plasma group was registered in the central region of the elevated space. This result agrees with that reported in a study mentioned above in which DBBM was used [24]. In that study, after 10 weeks of healing, a statistically significant difference was found in favor of the plasma group only for the central region. It might be speculated that, own to the low rate of resorption of the DBBM, a high content of bioactivated biomaterial was still present in this period of healing, yielding a higher bone formation in the plasma sites compared to the untreated control sites. It was concluded that the bio-activation of the DBBM increased bone formation in regions far from the osteogenic sources.

In the present study, the highest percentages of new bone were found in the bone walls region. This agrees with the outcomes reported by several experimental studies on sinus lifting [9–14], that showed that the most important source for bone formation is represented by the pre-exiting sinus bony walls. Instead, the role of the sinus membrane in the early phases of healing is still under debate [7–10, 32–37].

In conclusion, the bio-activation with argon plasma on a synthetic graft composed of HA and β-TCP used as filler material for sinus lifting showed a tendency to improve bone formation. This tendency was higher in the central region, far from the osteogenic sources. However, the difference between test and control sites was neither statistically significant nor clinically relevant. The properties of the synthetic biomaterial allowed new bone ingrowth into the graft, forming a network of bridges interconnecting the bone formed outside the granules with the remnants of the porous biomaterial.

A limitation of the present study was the limited sample and the loss of the specimen of one animal. The animal model used is another limitation due to its accelerate rate of healing compared to that in human [38]. Comparisons with biomaterial with a lower rate of resorption should be performed to disclose differences. Biopsies in humans should be performed to confirm the histological data, using similar synthetic biomaterial, and studying new bone formation around and inside the graft.

## Data Availability

The datasets used or analyzed during the current study are available from the corresponding author on reasonable request.
